# Correlation of 3D MRI‐Derived Adenohypophysis Volume With Height and Peak GH in Peripubertal Children

**DOI:** 10.1002/brb3.70922

**Published:** 2025-09-25

**Authors:** Jianjian Cai, Ling Zhang, Weiyin Vivian Liu, Qin Liu, Dong Liu, Wen Zhou

**Affiliations:** ^1^ Department of Radiology, Tongji Hospital, Tongji Medical College Huazhong University of Science and Technology Wuhan Hubei China; ^2^ Department of Urology, Tongji Hospital, Tongji Medical College Huazhong University of Science and Technology Wuhan Hubei China; ^3^ MR Research, GE Healthcare Beijing China

**Keywords:** adenohypophysis precise volume, diagnosis, height, maximum GH value, partial correlation

## Abstract

**Purpose:**

To investigate correlations between adenohypophysis volume (aPV) quantified by 3D CUBE T1 MRI, height, and peak GH levels (GH max), and evaluate its clinical utility.

**Materials and Methods:**

A total of 380 children (3–12 years) old with suspected growth and development disorders enrolled from May 2020 to July 2022. All of them underwent 3.0T pituitary MRI with 3D CUBE T1WI, serological testing, growth hormone stimulation testing, and aPV and peak GH levels were also measured. Pearson and partial correlation analysis assessed relationships between aPV, height, and peak GH.

**Results:**

Strong aPV correlations with weight (*r =* 0.603, *p < *0.001), age (*r =* 0.575, *p < *0.001), and height (*r =* 0.665, *p < *0.001) were observed in adolescent children. There was no significant correlation between aPV and peak GH (*r =* 0.083, *p =* 0.105), and between peak GH value and height (*r =* −0.108, *p =* 0.036) in adolescent children. No gender‐based aPV difference was observed using the Mann–Whitney *U* test (*p *> 0.05). After covariate adjustment (age, gender, weight), there were a significant aPV and height correlation (*r =* 0.250, *p < *0.001), a significant aPV and peak GH correlation (*r =* 0.246, *p < *0.001), and a significant peak GH and height correlation (*r =* 0.176, *p < *0.001).

**Conclusions:**

After controlling confounders, the correlation between the aPV and height exceeds the correlation between peak GH and height. aPV reflects not only GH secretion but also integrated endocrine regulation of growth thorough multiple hormones. 3D CUBE T1 volumetry provides a clinically valuable non‐invasive method for assessing growth and development disorders.

## Graphical abstract

This study found that the volume of the adenohypophysis has a strong correlation with height and has a certain weak correlation with GH maximum value. The volume of the adenophypophysis not only reflects the secretion of GH, but may also be closely related to the secretion of other hormones, playing a key role in height growth.

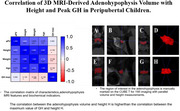

## 1 Background

The pituitary gland is the most important endocrine gland in the human body, divided into two parts: the anterior and posterior lobes. The anterior lobe (adenohypophysis) is primarily composed of glandular tissues and secretes various hormones that enter the systemic circulation to exert their effect. The posterior lobe (neurohypophysis) mainly consists of neural tissues and stores/releases two hormones produced by the hypothalamus. Adolescence is an important stage of growth and development. As a key component of the endocrine system, the adenohypophysis plays a core role in regulating the secretion of hormones such as growth hormone (GH). During children's development, pituitary size changes with age and weight (Çolaklar and Fitoz 2023; Fehrenbach et al. 2020; Liu, Liu, et al. 2024).

Children with short stature have significantly reduced adenohypophysis volume (PV), suggesting that PV is the cause of decreased GH secretion and poor growth (Sayegh et al. 2022). More severe GH deficiency correlates with smaller PV (*p =* 0.082) (Oh et al. 2024), indicating a close relationship between GH secretion and PV. GH treatment does not accelerate puberty onset but may prolong its duration without significantly affecting pubertal height gain (Zhu et al. 2024). In addition, two consecutive GH excitation tests can increase the growth rate in idiopathic short stature (ISS) (Tortora et al. 2024), demonstrating that GH secretion is closely related to height development. Compared to normal children, patients with isolated GH deficiency (GHD) showed the largest decrease in PV, while those with ISS also have decreased PV. The more severe the GH deficiency, the smaller the PV appears to be (*p =* 0.082) (Oh et al. 2024). However, compared to GHD patients, mean PV in ISS patients is significantly increased (Kiremitci Yilmaz et al. 2024). Magnetic resonance imaging (MRI) measurement of PV aids diagnostic evaluation in children with GHD (Kiremitci Yilmaz et al. 2024; Pellini et al. 1990; Kessler et al. 2016). These studies collectively indicate that PV is closely related to height growth.

GH secretion and the height and volume of the adenohypophysis are interconnected. However, the specific relationship between the volume and height of the adenohypophysis and peak GH (GHmax) in adolescents remains inadequately defined, and its role in height growth is unclear. Height growth is primarily regulated GH, with no other hormones playing an equally important role. This knowledge gap persists partly due to challenges in evaluating PV and difficulty in excluding confounding factors. Absolute or relative malnutrition reduces PV. In anorexia nervosa (AN) patients, PV positively correlates with body weight and luteinizing hormone (LH) level but negatively correlates with GH level (Merabet et al. 2024). Changes in pituitary gland size, especially in the childhood and adolescent stages, may be associated with the occurrence of certain mental illnesses. Factors such as age, gender, and hormone levels further affect the volume and function of Pituitary Gland (Anastassiadis et al. 2019). Subjects with post‐traumatic stress disorder (PTSD) have significantly larger PV than controls; however, PV was similar between prepubertal PTSD subjects and controls. PTSD subjects with a history of suicide ideation exhibited larger PV (Thomas and De Bellis 2004). With radiation‐free and high‐resolution characteristics, MRI is vital for evaluating adenohypophysis morphology and function. During children's development, the pituitary gland enlarges with age, height, and weight. Previous studies used 2D MRI images to measure pituitary height (superior‐inferior diameter), width (left‐right diameter), and length (anterior and posterior), inserting these into an ellipsoid model to estimate PV. However, pituitary morphology during the enlargement period is not ideally ellipsoidal, and inter‐individual shape variation means that size assessments can be highly inaccurate unless the true volume is measured. Advances in MRI, especially the widespread clinical use of 3D sequences, now enable accurate PV measurement. Nevertheless, the association mechanism between increased PV, GH secretion, and height growth is confounded by multiple factors, consequently leading to serious underestimation of PV's potential diagnostic value.

This study aims to measure the true adenohypophysis volume using a 3D CUBE sequence and analyze its association with gender, weight, age, height, and GHmax. It further seeks to explore the mechanism underlying the growth of adenohypophysis volume and height in early adolescence and adolescent children.

## 2 Materials and Methods

This study was approved by the Institutional Review Boards (IRB) of our hospital (IRB No. TJ‐RB202303157).

### 2.1 Clinical Case Collection

This study included 380 children (aged 3–12 years old) with clinically suspected growth and development diseases who were admitted to our hospital from May 2020 to July 2022. The inclusion criteria are as follows: (1) completion of a 3.0 T pituitary MRI using CUBE T1‐weighted imaging (T1WI) and (2) performance of serological testing within 2 days before and after the MRI scan, preferably in the early morning. Exclusion criteria are as follows: (1) MR examinations with significant motion artifacts or other factors compromising image quality; (2) conditions potentially affecting GHRH‐GH‐IGF (hypothalamic‐growth hormone‐insulin‐like growth factor) axis, including secondary causes such as hypothalamus, pituitary or other intracranial tumors, infection, cell infiltration, radiation damage and head trauma, etc.; (3) history of hormone therapy, neurofibroma or congenital adrenal hyperplasia, etc.;(4)morphologically abnormal pituitary gland configuration (e.g., significant convexity/raised appearance). Ultimately, 380 children were included in the study cohort.

### 2.2 General Clinical Data Collection

Patient's weight, height, age, and serology test results were collected. Serological analysis included measurement of maximum GH level.

### 2.3 GH Excitation Experiment

Maximum GH levels were measured by chemiluminescence immuno‐assay. Fasting serum samples were collected at 9 a.m. clonidine was then administered intravenously at a dose of 0.5–1.0 g/kg body weight, followed by serial blood sampling at an interval of 15–30 min to measure GH concentration in the blood. Three to five samples were typically collected until peak stimulation response or predetermined endpoint attainment.

### 2.4 MRI Examination and MRI Vision Assessment

MRI protocols were performed per our previously published methodology [JMRI and CR]. Pituitary MRI was performed using a 3.0T scanner (Discovery 750; GE Healthcare, USA) using a 32‐channel head coil. Sagittal 3D CUBE T1WI sequence parameters are as follows: TR/TE = 600/14 ms; slice thickness = 0.8 mm; echo chain = 24; matrix = 256 × 192; FOV = 200 mm × 200 mm^;^ voxel = 0.3906 mm × 0.3906 mm × 0.8 mm; bandwidth = 41.67 Hz; NEX = 1; acquisition time = 1 min 47 s.

### 2.5 MRI Vision Assessment

**FIGURE 1:**
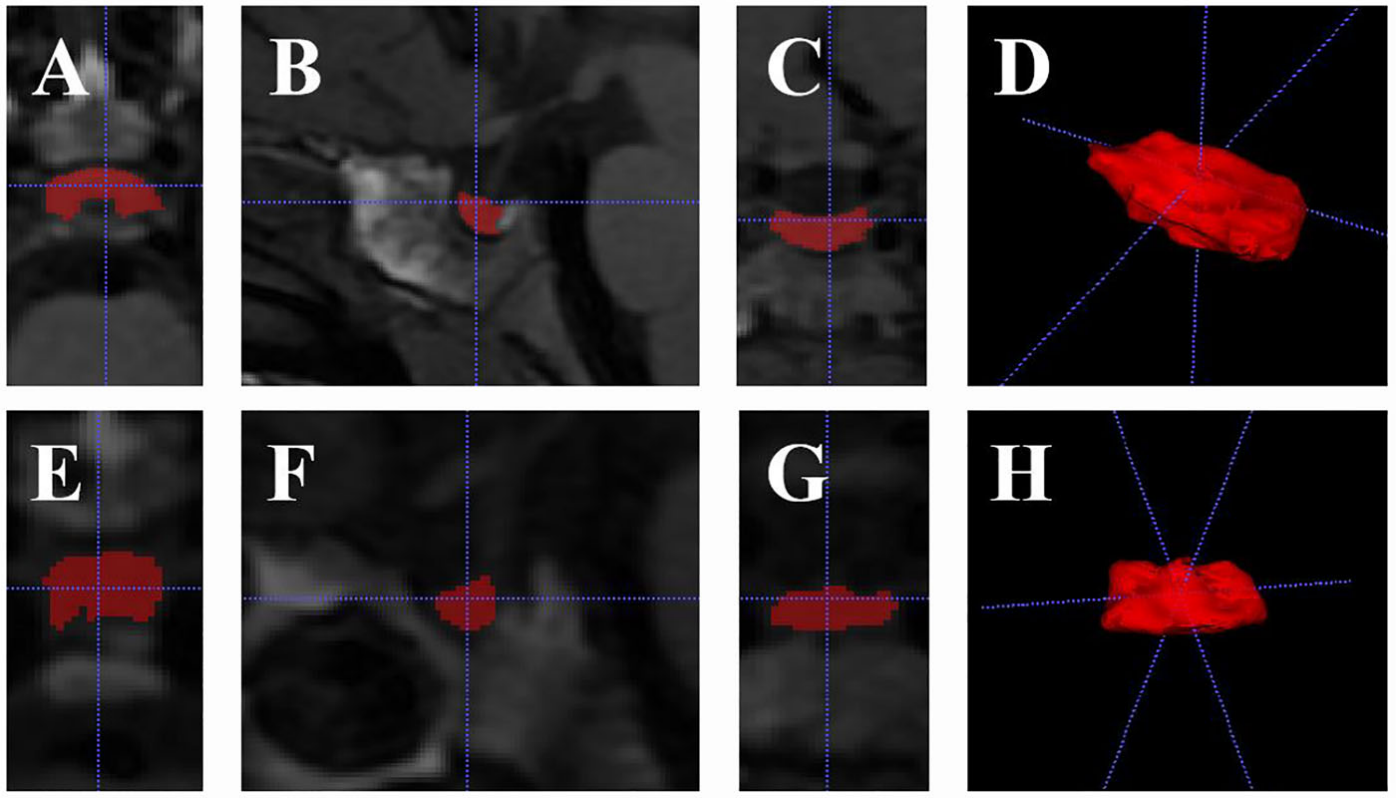
Manual segmentation of adenohypophysis on CUBE T1WI with volume and height measurements. Case 1: Female, age = 5.12 years, weight = 16 kg, height = 101.6 cm, peak GH = 4.82 mU/mL, aPV = 325.40 mm^3^. (A–C) Axial, sagittal, and coronal ROI delineation; (D) 3D volume reconstruction. Case 2: Female, age = 4.45 years, weight = 16 kg, height = 100 cm, peak GH = 16.50 mU/mL, aPV = 107.90 mm^3^. (E–G) Axial, sagittal, and coronal ROI delineation; (H) 3D volume reconstruction.

**FIGURE 2:**
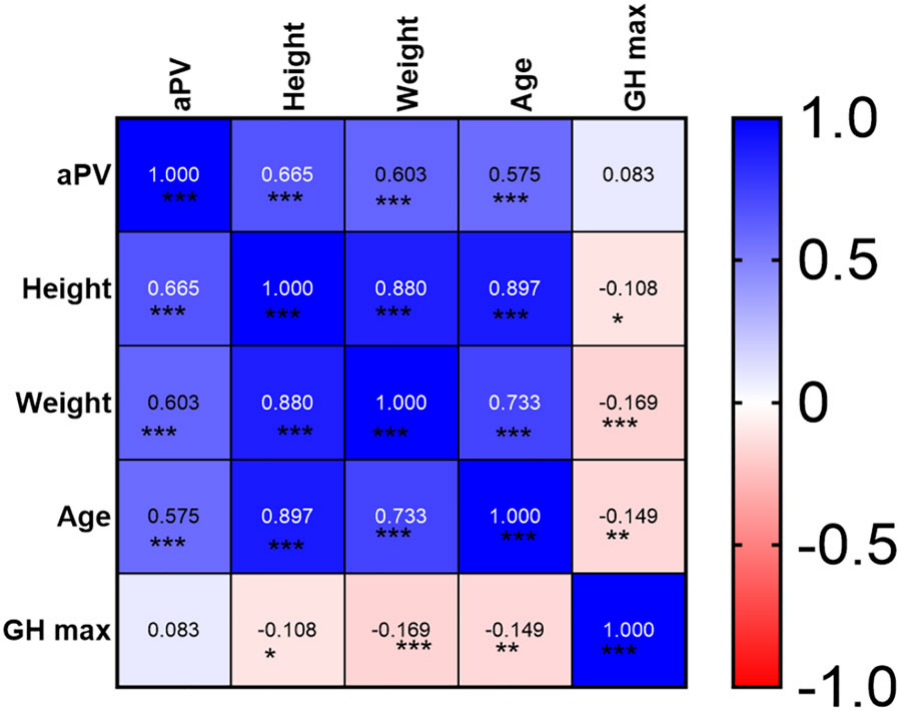
Correlation matrix of characteristics, adenohypophysis MRI features, and biochemical indicators.

**FIGURE 3:**
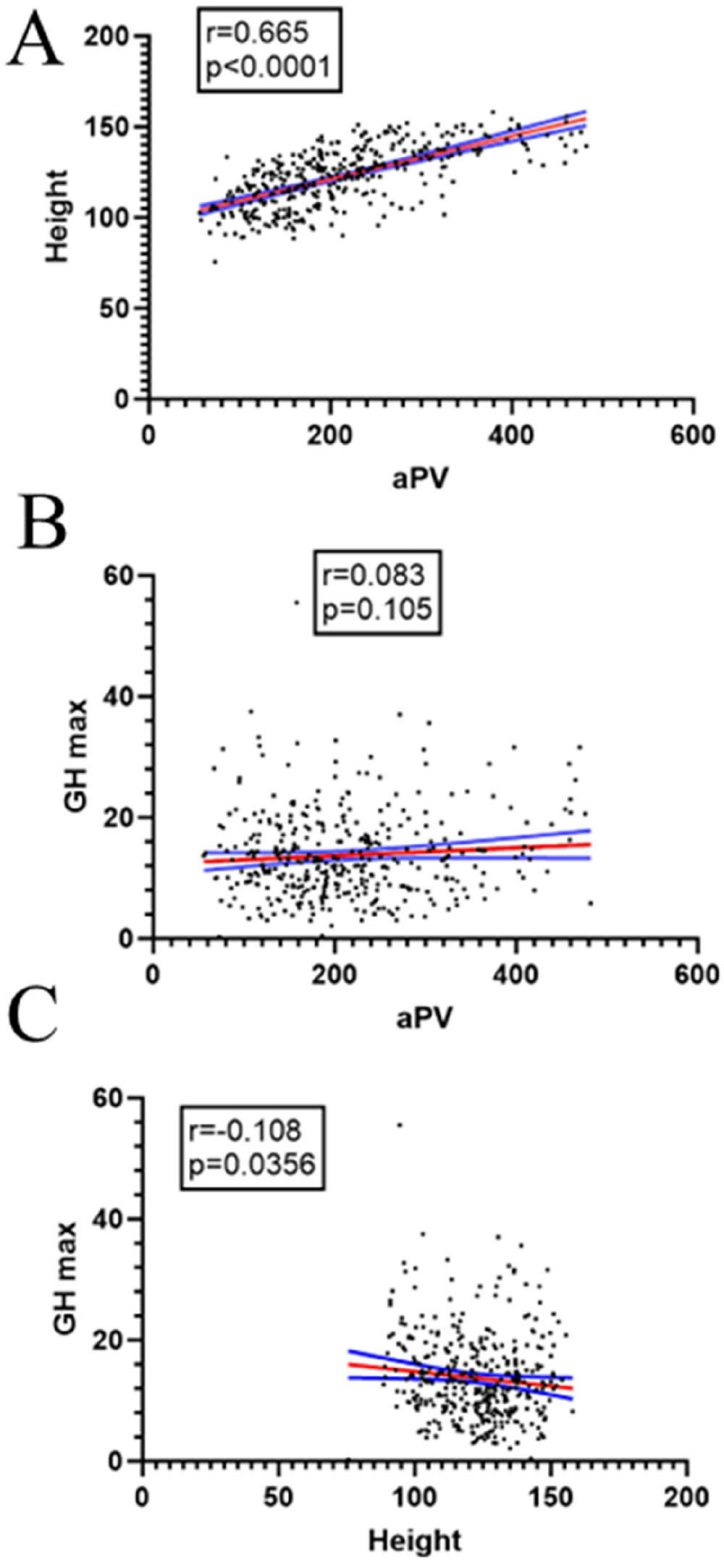
Scatter plots of aPV correlations with height and peak GH. (A) aPV–height correlation. (B) aPV–peak GH correlation. (C) Height–peak GH correlation.

Two neuroradiologists (with 15 and 7 years of experience in pituitary MRI, respectively) retrospectively and independently reviewed all scans. Patients with pituitary abnormalities, such as pituitary stalk disruption, Rathke's cleft cyst, pituitary tumors and motion artifacts, and poor image quality, were excluded. ITK‐SNAP (version 2003.8.0, http://www.itksnap.org/pmwiki/pmwiki.php) was used to measure the MRI volume (aPV) and midsagittal height (aPH) of the adenohypophysis. The outline and measurement of the adenohypophysis and boundary definition are referred (Liu, Lv, et al. 2024; Satogami et al. 2010) (Figure 1). All segmentation underwent confirmation by a senior radiologist; discrepancies were resolved by consensus.(Figure 2 and Figure 3)

### 2.6 Statistical Analysis

Statistical analysis was performed using SPSS (version 22.0, SPSS Inc., Chicago). Data are expressed as mean ± standard deviation (SD). Pearson and partial correlation analysis assessed relationships between volume, height, weight, age, and peak GH concentration of adenohypophysis. Two independent samples’ non‐parametric tests (i.e., Mann–Whitney *U* test) analyzed gender differences in adenohypophysis volume.Table 1

## 3 Results

### 3.1 Clinical Cohort Characteristics

**TABLE 1:** Demographic, adenohypophysis MRI, and biochemical characteristics of the study cohort.

Characteristics	(mean ± SD)
*N*	380
age	7.67 ± 0.49
Height, cm	122.38 ± 16.23
HtSDS	1.17 ± 1.07
Weight, kg	25.59 ± 9.69
BMI	17.18 ± 3.22
aPH, mm	4.05 ± 1.05
aPV, mm^3^	211.03 ± 90.79
Gender (male:female)	1:1.26
GH max, mU/mL	13.75 ± 7.21

*Note*: Measurement data were analyzed using *t‐*test.

Abbreviations: aPH: adenohypophysis height, aPV: adenohypophysis volume, BMI: body mass index, GH max: peak GH, HtSDS: height standard deviation score.

This retrospective analysis included 380 children (mean age: 7.67 ± 0.49; range: 3.24–14.64 years), comprising 168 males and 212 females. Mean peak GH (GH max) was 13.75 ± 7.21 mU/mL, with 253 children (male: 110, female: 143 cases) showing GH ≥ 10 mU/mL and 127 children (male: 58 cases, female: 69 cases) with GH < 10. Mean height was 122.38 ± 16.23 cm(Table 1).

### 3.2 Correlation Between Adenohypophysis Volume and Weight, Age, and Gender

Pearson correlation analysis revealed strong correlations between aPV and weight (*r =* 0.603, *p < *0.001) and age (*r =* 0.575, *p < *0.001). Mann–Whitney *U* test showed no significant gender‐based aPV difference (*p* > 0.05)(Figure 2).

### 3.3 Correlation Between Adenohypophysis Volume and Height

Strong positive correlation existed between aPV and height in adolescent children (*r =* 0.665, *p < *0.001). After controlling for age, gender, and weight via partial correlation, significant weak positive correlation persisted (*r =* 0.250, *p < *0.001)(Figure 3A).

### 3.4 Correlation Between Adenohypophysis Volume and GH Maximum Value

No significant correlation was observed between aPV and peak GH in adolescent children (*r =* 0.083, *p =* 0.105). After controlling for age, gender, and weight, there was a significant weak positive correlation (*r =* 0.246, *p < *0.001)(Figure 3B).

### 3.5 Correlation Between GH Maximum Value and Height

No significant correlation existed between peak GH and height in adolescent children (*r =* −0.108, *p =* 0.036). After controlling for age, gender, and weight, there was a significant weak correlation between aPV and height (*r =* 0.176, *p < *0.001)(Figure 3C).

## 4 Discussion

This study systematically analyzes relationships between aPV and GH secretion, and physical development in children, revealing three important findings: (1) aPV shows significantly positive correlations with weight (*r =* 0.603), age (*r =* 0.575), and height (*r =* 0.665), with no gender association. (2) After controlling for confounders, aPV and height maintain independent correlation (partial *r =* 0.250, *p < *0.001). (3) The peak GH–height correlation strength (partial *r =* 0.176) is significantly lower than aPV–height correlation. These results suggested that adenohypophysis morphological development has biological significance in growth regulation extending beyond GH secretion alone.

### 4.1 Developmental Characteristics of Adenohypophysis Volume

Consistent with Lurie et al. (1990), aPV showed age‐dependent progression. Notably, the correlation between aPV and height (*r =* 0.665) exceeds correlations with weight (*r =* 0.603) and age (*r =* 0.575), suggesting synchronized adenohypophysis development and linear growth. Height regulation involves not only GH but also multiple hormones, modulating the activity of chondrocytes on the growth plate (e.g., thyroid‐stimulating hormone [TSH]) and follicle stimulating hormone [FSH]) (Baron et al. 2015). As the adenohypophysis secretes and regulates GH, TSH, FSH, and related hormones, it provides a biological basis for volume–height correlation.

### 4.2 Re‐Evaluation of Volume‐Functional Relationships

Traditional views suggest limited diagnostic value of adenohypophysis volumetry in growth disorders (Oh et al. 2024; Akkus et al. 2021). However, our study demonstrated significantly strengthened correlation between aPV and peak GH after controlling for confounding factors (*r =* 0.083→0.246), indicating a potential underestimation of the functional significance of the adenohypophysis structural development. Additionally, the pituitary morphology is categorized into flat, convex, and concave configurations, demonstrating shape changes with height and age (Bonczar et al. 2023). ITK‐SNAP enables manual segmentation with precise anatomical boundary delineation—particularly valuable in complex neuroanatomical regions. This approach provides superior accuracy versus automated methods (Ali et al. 2022). While adenohypophysis morphological heterogeneity causes volumetric deviations (Zennadi et al. 2024), our ITK‐SNAP manual segmentation protocol enhances measurement precision (Lindberg et al. 2018). These findings support combined imaging volumetry and dynamic hormone testing in clinical growth disorder assessment.

### 4.3 Multi‐Hormonal Synergy

The maintained aPV–height correlation (partial *r =* 0.250) significantly exceeds the peak GH–height relationship (partial *r =* 0.176), suggesting that adenohypophysis‐mediated growth regulation involves multiple hormones. The adenohypophysis volume insufficiently distinguishes between GH deficiency and idiopathic shortness (Oh et al. 2024). Moreover, the strong aPV–height correlation revealed a theoretical basis for developing new differential diagnostic indicators.

### 4.4 Limitations

This study has several limitations: (1) The cross‐sectional nature precludes causal inference. (2) Downstream effectors (e.g., IGF‐1) were not assessed. (3) Subgroup analysis depth was constrained by sample size. Future longitudinal cohorts should track aPV developmental trajectories, integrated with single‐cell sequencing to analyze volume‐cell differentiation relationships. For clinical transformation, we recommend incorporating aPV into routine evaluation system for growth retardation, alongside exploration of its combined diagnostic value with GH stimulation testing.

## 5 Conclusions

Adenohypophysis volume demonstrates a strong correlation with height, significant (though weaker) correlation with peak GH after covariate adjustment, and associations not limited to GH secretion but potentially involving multiple hormones mediating growth regulation. These findings support pituitary volumetry as a clinically valuable, non‐invasive method for evaluating growth and developmental diseases.

## Author Contributions

**Jianjian Cai**: writing–original draft, writing–review and editing, data curation. **Ling Zhang**: conceptualization, methodology. **Weiyin Vivian Liu**: investigation, validation. **Qin Liu**: supervision, funding acquisition, visualization. **Dong Liu**: project administration, resources. **Wen Zhou**: software, formal analysis.

## Peer Review

The peer review history for this article is available at https://publons.com/publon/10.1002/brb3.70922.

## Data Availability

The data that support the findings of this study are available from the corresponding author upon reasonable request.

## References

[brb370922-bib-0001] Akkus, G. , S. Sözütok , F. Odabaş , et al. 2021. “Pituitary Volume in Patients With Primary Empty Sella and Clinical Relevance to Pituitary Hormone Secretion: A Retrospective Single Center Study.” Current Medical Imaging 17, no. 8: 1018–1024. 34036923 10.2174/1573405617666210525111218 PMC8653417

[brb370922-bib-0002] Ali, M. , J. Su Suh , M. Ramonas , et al. 2022. “A Detailed Manual Segmentation Procedure for the Hypothalamus for 3T T1‐Weighted MRI.” MethodsX 9: 101864. 36193115 10.1016/j.mex.2022.101864 PMC9526169

[brb370922-bib-0003] Anastassiadis, C. , S. L. Jones , and J. C. Pruessner . 2019. “Imaging the Pituitary in Psychopathologies: A Review of In Vivo Magnetic Resonance Imaging Studies.” Brain Structure & Function 224, no. 8: 2587–2601. 31432271 10.1007/s00429-019-01942-5

[brb370922-bib-0004] Baron, J. , L. Sävendahl , F. De Luca , et al. 2015. “Short and Tall Stature: A New Paradigm Emerges.” Nature Reviews Endocrinology 11, no. 12: 735–746. 10.1038/nrendo.2015.165 PMC5002943 26437621

[brb370922-bib-0005] Bonczar, M. , G. Wysiadecki , P. Ostrowski , et al. 2023. “The Morphology of the Pituitary Gland: A Meta‐Analysis With Implications for Diagnostic Imaging.” Brain Sciences 13, no. 1: 89. 36672070 10.3390/brainsci13010089 PMC9856875

[brb370922-bib-0006] Çolaklar, A. , and Ö. S. Fitoz . 2023. “Pituitary Gland Volumes in Children With Normal Endocrine Function.” Pediatric Radiology 53, no. 3: 450–460. 36138218 10.1007/s00247-022-05505-5

[brb370922-bib-0007] Fehrenbach, U. , A. Jadan , T. A. Auer , et al. 2020. “Obesity and Pituitary Gland Volume—A Correlation Study Using Three‐Dimensional Magnetic Resonance Imaging.” Neuroradiology Journal 33, no. 5: 400–409. 32666872 10.1177/1971400920937843 PMC7482044

[brb370922-bib-0008] Kessler, M. , M. Tenner , M. Frey , and R. Noto . 2016. “Pituitary Volume in Children With Growth Hormone Deficiency, Idiopathic Short Stature and Controls.” Journal of Pediatric Endocrinology & Metabolism 29, no. 10: 1195–1200. 27710916 10.1515/jpem-2015-0404

[brb370922-bib-0009] Kiremitci Yilmaz, S. , G. Yilmaz Ovali , D. Ozalp Kizilay , S. Tarhan , and B. Ersoy . 2024. “Pitfalls of Diagnosing Pituitary Hypoplasia in the Patients With Short Stature.” Endocrine 86, no. 1: 349–357. 38969909 10.1007/s12020-024-03951-9 PMC11445333

[brb370922-bib-0010] Lindberg, K. , A. Kouti , D. Ziegelitz , T. Hallén , T. Skoglund , and D. Farahmand . 2018. “Three‐Dimensional Volumetric Segmentation of Pituitary Tumors: Assessment of Inter‐Rater Agreement and Comparison With Conventional Geometric Equations.” Journal of Neurological Surgery 79, no. 5: 475–481. 30210975 10.1055/s-0037-1618577 PMC6133660

[brb370922-bib-0011] Liu, D. , W. V. Liu , L. Zhang , et al. 2024. “Diagnostic Value of Adenohypophyseal MRI Features in Female Children With Precocious Puberty.” Clinical Radiology 79, no. 3: 179–188. 38114375 10.1016/j.crad.2023.11.020

[brb370922-bib-0012] Liu, D. , W. Lv , W. V. Liu , et al. 2024. “MRI Radiomics Features of Adenohypophysis Determine the Activation of Hypothalamic‐Pituitary‐Gonadal Axis in Peri‐Puberty Children.” Journal of Magnetic Resonance Imaging 59, no. 5: 1769–1776. 37501392 10.1002/jmri.28914

[brb370922-bib-0013] Lurie, S. N. , P. M. Doraiswamy , M. M. Husain , et al. 1990. “In Vivo Assessment of Pituitary Gland Volume With Magnetic Resonance Imaging: The Effect of Age.” Journal of Clinical Endocrinology and Metabolism 71, no. 2: 505–508. 2380345 10.1210/jcem-71-2-505

[brb370922-bib-0014] Merabet, M. , N. Germain , J. Redouté , et al. 2024. “Structure‐Function Relationship of the Pituitary Gland in Anorexia Nervosa and Intense Physical Activity.” Brain Structure & Function 229, no. 1: 195–205. 38062204 10.1007/s00429-023-02739-3

[brb370922-bib-0015] Oh, J. S. , B. Sohn , Y. Choi , et al. 2024. “The Influence of Pituitary Volume on the Growth Response in Growth Hormone‐Treated Children With Growth Hormone Deficiency or Idiopathic Short Stature.” Annals of Pediatric Endocrinology & Metabolism 29, no. 2: 95–101. 37946439 10.6065/apem.2346052.026 PMC11076225

[brb370922-bib-0016] Pellini, C. , B. di Natale , R. De Angelis , et al. 1990. “Growth Hormone Deficiency in Children: Role of Magnetic Resonance Imaging in Assessing Aetiopathogenesis and Prognosis in Idiopathic Hypopituitarism.” European Journal of Pediatrics 149, no. 8: 536–541. 2112091 10.1007/BF01957687

[brb370922-bib-0017] Satogami, N. , Y. Miki , T. Koyama , M. Kataoka , and K. Togashi . 2010. “Normal Pituitary Stalk: High‐Resolution MR Imaging at 3T.” American Journal of Neuroradiology 31, no. 2: 355–359. 19797792 10.3174/ajnr.A1836 PMC7964128

[brb370922-bib-0018] Sayegh, E. , L. McGuirk , T. Patale , et al. 2022. “PSAT107 Evaluating Pituitary Volume and the Growth Hormone Stimulation Test in Siblings. Both Together Better Define the Etiology of Short Stature.” Supplement, Journal of the Endocrine Society 6, no. S1: A633–A634.

[brb370922-bib-0020] Thomas, L. A. , and M. D. De Bellis . 2004. “Pituitary Volumes in Pediatric Maltreatment‐Related Posttraumatic Stress Disorder.” Biological Psychiatry 55, no. 7: 752–758. 15039005 10.1016/j.biopsych.2003.11.021

[brb370922-bib-0021] Tortora, A. , V. Marotta , G. Izzo , D. Rocco , G. Clemente , and M. Vitale . 2024. “GH Provocative Tests Stimulate the Growth in Children With Idiopathic Short Stature.” Endocrine 85, no. 2: 849–854. 38750401 10.1007/s12020-024-03860-x PMC11291594

[brb370922-bib-0022] Zennadi, M. M. , M. Ptito , J. Redouté , et al. 2024. “MRI Atlas of the Pituitary Gland in Young Female Adults.” Brain Structure & Function 229, no. 4: 1001–1010. 38502330 10.1007/s00429-024-02779-3

[brb370922-bib-0023] Zhu, F. , Q. Xu , L. Huang , J. Zhu , L. Huang , and Y. Zhang . 2024. “Effects of Growth Hormone Therapy on the Onset and Progression of Pubertal Development in Girls With Idiopathic Short Stature.” Gynecological Endocrinology 40, no. 1: 2358227. 38807420 10.1080/09513590.2024.2358227

